# Supplementary figures and images for: Trends in bacterial and fungal communities in ant nests observed with Terminal-Restriction Fragment Length Polymorphism (T-RFLP) and Next Generation Sequencing (NGS) techniques—validity and compatibility in ecological studies
Source: PeerJ. 2018 Jul 20;6:e5289. doi: 10.7717/peerj.5289 (PMC6055595; doi:10.7717/peerj.5289)

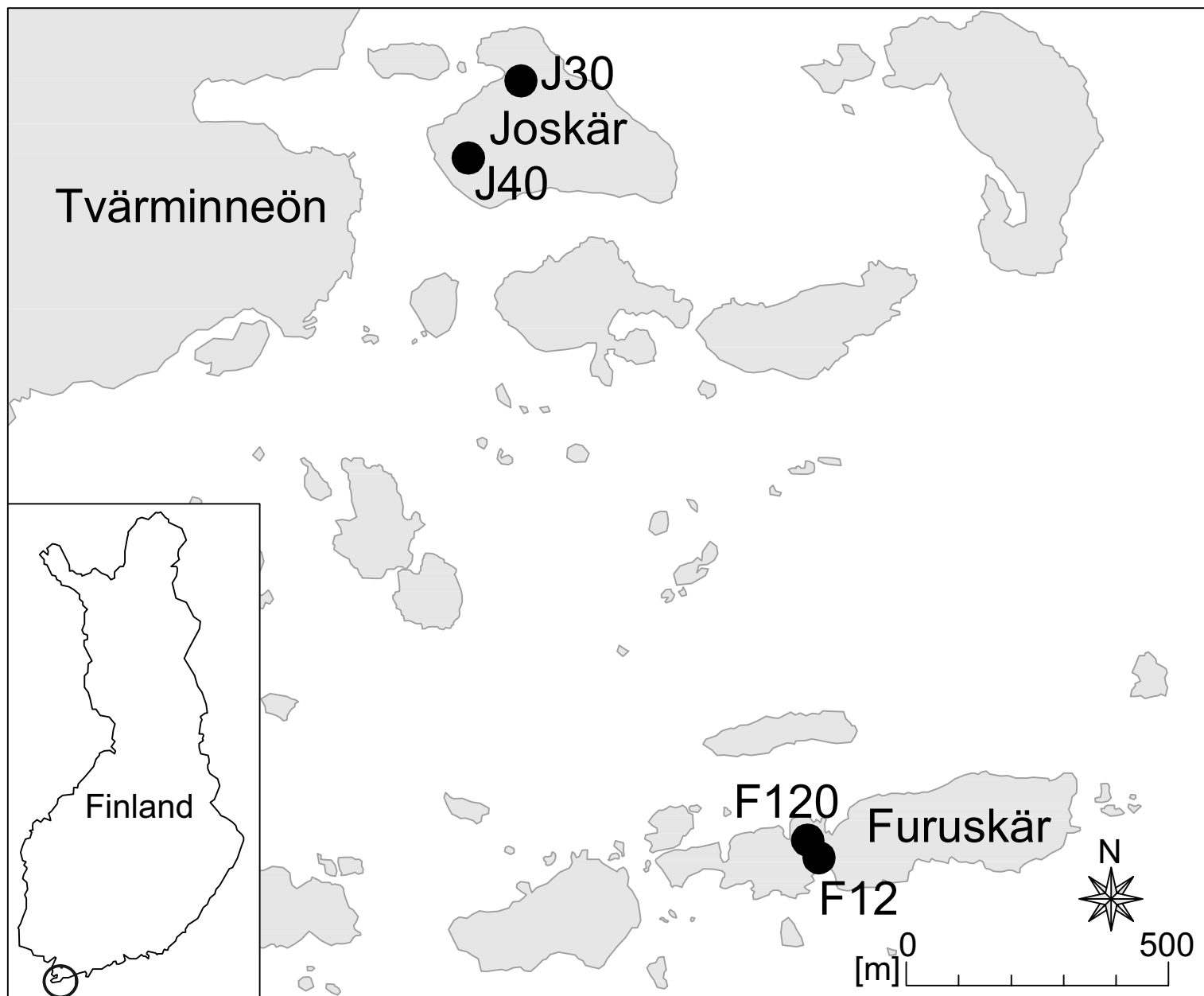

Supplement: Figure S1 [file peerj-06-5289-s001.pdf]

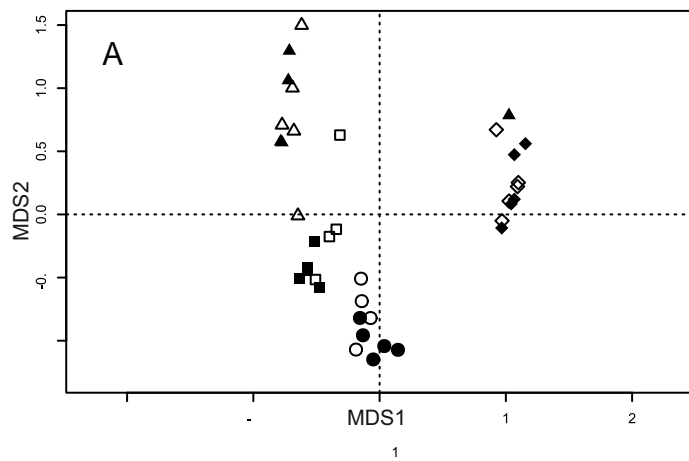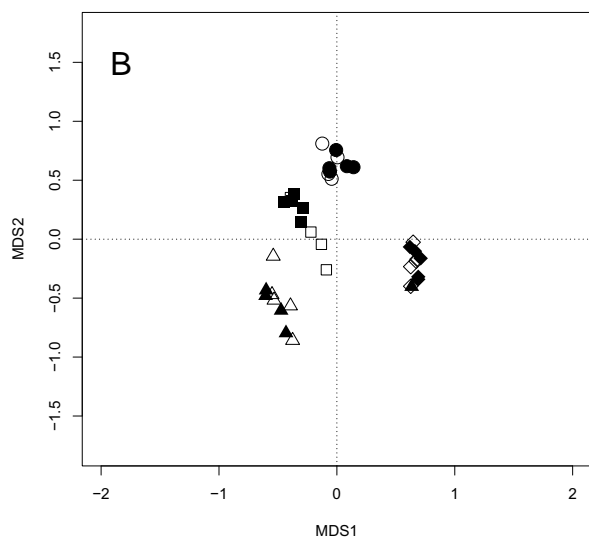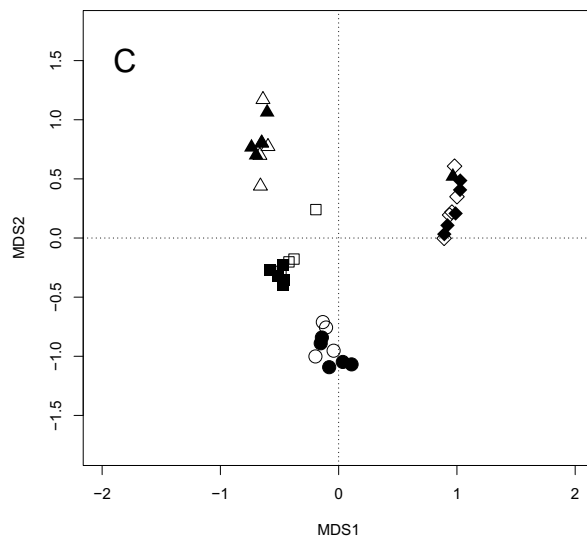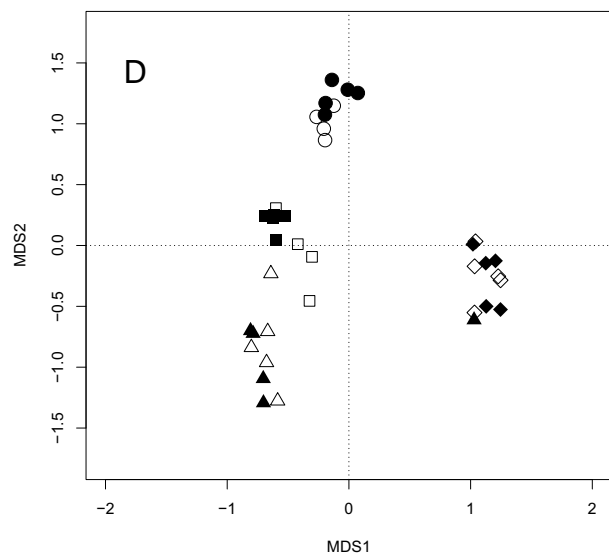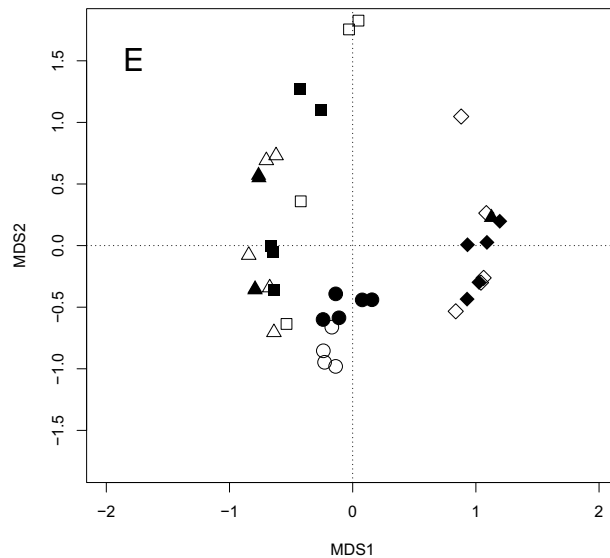

Supplement: Figure S2 — Bray-Curtis distance of non-transformed (A), square root (B) and log-normal (C) transformed data, and of Jaccard distances (D) and Morisita distances (E) on non-transformed data. [file peerj-06-5289-s002.pdf]
