# Supplementary material for: Trends in bacterial and fungal communities in ant nests observed with Terminal-Restriction Fragment Length Polymorphism (T-RFLP) and Next Generation Sequencing (NGS) techniques—validity and compatibility in ecological studies
Source: PeerJ. 2018 Jul 20;6:e5289. doi: 10.7717/peerj.5289 (PMC6055595; doi:10.7717/peerj.5289)
Supplement: Table S1 — Parameters for the pipeline: Merge: -fastq_maxdiffs 10 -fastq_pctid 10 -fastq_minmergelen 100 Filter: -fastq_maxee 1.0 Dereplicate: -minuniquesize 2 Note that the number of reads in the OTU table is larger than the number of filtered reads; this is because all merged reads are aligned to the OTUs to create the OTU table (https://www.drive5.com/usearch/manual/). [file peerj-06-5289-s005.docx]

|  | **Bacteria** | **Fungi** |
| --- | --- | --- |
| Total reads (R1+R2) | 7489836 | 8833960 |
| Merged reads | 2614533 | 3487738 |
| Filtered reads | 1241782 | 968852 |
| Reads in OTU table | 1933492 | 3368793 |
| Reads (singletons and doubletons removed) | 1896920 | 3274825 |
| Mean no. of reads / sample (*S.D.*) | 49949 (13406) | 86180 (31683) |
| Lowest no. of reads / sample | 22616 | 25002 |
