# Supplementary material for: Trends in bacterial and fungal communities in ant nests observed with Terminal-Restriction Fragment Length Polymorphism (T-RFLP) and Next Generation Sequencing (NGS) techniques—validity and compatibility in ecological studies
Source: PeerJ. 2018 Jul 20;6:e5289. doi: 10.7717/peerj.5289 (PMC6055595; doi:10.7717/peerj.5289)
Supplement: Table S2 [file peerj-06-5289-s006.pdf]

Supplementary Table S2. Bacteria – the 93 virtually restricted OTUs matched with T-RFs

Restr pattern	Phylum	Class	Order	Family
1	Actinobacteria	Actinobacteria	Actinomycetales	Nocardioidaceae
1	Actinobacteria	Actinobacteria	Actinomycetales	Nocardioidaceae
1	Actinobacteria	Actinobacteria	Actinomycetales	Propionibacteriaceae
2	Proteobacteria	Alphaproteobacteria	Sphingomonadales	Sphingomonadaceae
3	Actinobacteria	Actinobacteria	Actinomycetales	
3	Actinobacteria	Actinobacteria	Actinomycetales	
3	Actinobacteria	Actinobacteria	Actinomycetales	
4	Actinobacteria	Actinobacteria	Actinomycetales	Streptomycetaceae
4	Actinobacteria	Actinobacteria	Actinomycetales	Streptomycetaceae
5	Actinobacteria	Actinobacteria	Actinomycetales	Microbacteriaceae
5	Actinobacteria	Actinobacteria	Actinomycetales	Streptomycetaceae
5	Actinobacteria	Actinobacteria	Actinomycetales	Streptomycetaceae
5	Actinobacteria	Actinobacteria	Actinomycetales	
5	Actinobacteria	Actinobacteria	Actinomycetales	Microbacteriaceae
6	Actinobacteria	Actinobacteria	Solirubrobacterales	-
7	Proteobacteria	Betaproteobacteria	Burkholderiales	Burkholderiaceae
7	Proteobacteria	Betaproteobacteria	Burkholderiales	Oxalobacteraceae
7	Proteobacteria	Betaproteobacteria	Burkholderiales	Oxalobacteraceae
7	Proteobacteria	Betaproteobacteria	Burkholderiales	Oxalobacteraceae
7	Proteobacteria	Betaproteobacteria	Burkholderiales	Oxalobacteraceae
7	Proteobacteria	Betaproteobacteria	Burkholderiales	Oxalobacteraceae
8	Proteobacteria	Betaproteobacteria	Burkholderiales	Burkholderiaceae
8	Proteobacteria	Betaproteobacteria	Burkholderiales	Burkholderiaceae
8	Proteobacteria	Betaproteobacteria	Burkholderiales	Burkholderiaceae
9	Proteobacteria	Betaproteobacteria	Burkholderiales	Burkholderiaceae
9	Proteobacteria	Betaproteobacteria	Burkholderiales	Burkholderiaceae
9	Proteobacteria	Betaproteobacteria	Burkholderiales	Burkholderiaceae
9	Actinobacteria	Actinobacteria	Actinomycetales	
9	Proteobacteria	Betaproteobacteria	Burkholderiales	
9	Proteobacteria			
9	Proteobacteria			
10	Actinobacteria	Actinobacteria	Actinomycetales	Pseudonocardiaceae
10	Actinobacteria	Actinobacteria	Actinomycetales	Pseudonocardiaceae
10	Actinobacteria	Actinobacteria	Actinomycetales	
10	Actinobacteria	Actinobacteria	Actinomycetales	
10	Actinobacteria	Actinobacteria	Actinomycetales	
10	Actinobacteria	Actinobacteria	Actinomycetales	
10	Actinobacteria	Actinobacteria	Actinomycetales	
10	Proteobacteria	Alphaproteobacteria	Rhizobiales	
11	Proteobacteria	Alphaproteobacteria	Rhizobiales	Beijerinckiaceae
11	Proteobacteria	Alphaproteobacteria	Rhizobiales	Bradyrhizobiaceae
11	Proteobacteria	Alphaproteobacteria	Rhizobiales	Bradyrhizobiaceae
11	Proteobacteria	Alphaproteobacteria	Rhizobiales	Bradyrhizobiaceae
11	Proteobacteria	Alphaproteobacteria	Caulobacterales	Caulobacteraceae
11	Proteobacteria	Alphaproteobacteria	Caulobacterales	Caulobacteraceae

11	Proteobacteria	Alphaproteobacteria	Caulobacterales	Caulobacteraceae
11	Proteobacteria	Alphaproteobacteria	Caulobacterales	Caulobacteraceae
11	Proteobacteria	Alphaproteobacteria	Caulobacterales	Caulobacteraceae
11	Proteobacteria	Alphaproteobacteria	Caulobacterales	Caulobacteraceae
11	Proteobacteria	Alphaproteobacteria	Caulobacterales	Caulobacteraceae
11	Proteobacteria	Alphaproteobacteria	Caulobacterales	Caulobacteraceae
11	Proteobacteria	Alphaproteobacteria	Sphingomonadales	Erythrobacteraceae
11	Actinobacteria	Actinobacteria	Actinomycetales	Geodermatophilaceae
11	Proteobacteria	Alphaproteobacteria	Rhizobiales	Methylobacteriaceae
11	Proteobacteria	Alphaproteobacteria	Rhizobiales	Methylobacteriaceae
11	Proteobacteria	Alphaproteobacteria	Rhizobiales	Methylobacteriaceae
11	Actinobacteria	Actinobacteria	Actinomycetales	Mycobacteriaceae
11	Actinobacteria	Actinobacteria	Actinomycetales	Mycobacteriaceae
11	Actinobacteria	Actinobacteria	Actinomycetales	Mycobacteriaceae
11	Actinobacteria	Actinobacteria	Actinomycetales	Mycobacteriaceae
11	Actinobacteria	Actinobacteria	Actinomycetales	Mycobacteriaceae
11	Actinobacteria	Actinobacteria	Actinomycetales	Mycobacteriaceae
11	Actinobacteria	Actinobacteria	Actinomycetales	Mycobacteriaceae
11	Actinobacteria	Actinobacteria	Actinomycetales	Pseudonocardiaceae
11	Actinobacteria	Actinobacteria	Actinomycetales	Pseudonocardiaceae
11	Actinobacteria	Actinobacteria	Actinomycetales	Pseudonocardiaceae
11	Actinobacteria	Actinobacteria	Actinomycetales	Pseudonocardiaceae
11	Acidobacteria	Acidobacteria_Gp1		
11	Actinobacteria	Actinobacteria	Actinomycetales	
11	Proteobacteria	Alphaproteobacteria		
12	Proteobacteria	Alphaproteobacteria	Caulobacterales	Caulobacteraceae
12	Proteobacteria	Alphaproteobacteria	Caulobacterales	Caulobacteraceae
12	Proteobacteria	Alphaproteobacteria	Caulobacterales	Caulobacteraceae
12	Proteobacteria	Alphaproteobacteria	Caulobacterales	Caulobacteraceae
12	Proteobacteria	Alphaproteobacteria	Caulobacterales	Caulobacteraceae
13	Actinobacteria	Actinobacteria	Actinomycetales	Nocardioidaceae
14	Actinobacteria	Actinobacteria	Actinomycetales	Nocardioidaceae
14	Actinobacteria	Actinobacteria	Actinomycetales	
14	Actinobacteria	Actinobacteria	Actinomycetales	
15	Actinobacteria	Actinobacteria	Actinomycetales	Microbacteriaceae
15	Actinobacteria	Actinobacteria	Actinomycetales	Microbacteriaceae
15	Actinobacteria	Actinobacteria	Actinomycetales	Microbacteriaceae
15	Actinobacteria	Actinobacteria	Actinomycetales	Micromonosporaceae
15	Actinobacteria	Actinobacteria	Actinomycetales	Pseudonocardiaceae
15	Actinobacteria	Actinobacteria	Actinomycetales	Streptomycetaceae
15	Actinobacteria	Actinobacteria	Actinomycetales	Streptomycetaceae
15	Actinobacteria	Actinobacteria	Actinomycetales	Streptomycetaceae
15	Actinobacteria	Actinobacteria	Actinomycetales	Streptomycetaceae
15	Actinobacteria	Actinobacteria	Actinomycetales	Streptomycetaceae
15	Actinobacteria	Actinobacteria	Actinomycetales	
16	Acidobacteria	Acidobacteria_Gp1		
16	Acidobacteria	Acidobacteria_Gp1		
17	Actinobacteria	Actinobacteria	Actinomycetales	Microbacteriaceae
