# Supplementary material for: Trends in bacterial and fungal communities in ant nests observed with Terminal-Restriction Fragment Length Polymorphism (T-RFLP) and Next Generation Sequencing (NGS) techniques—validity and compatibility in ecological studies
Source: PeerJ. 2018 Jul 20;6:e5289. doi: 10.7717/peerj.5289 (PMC6055595; doi:10.7717/peerj.5289)
Supplement: Table S3 [file peerj-06-5289-s007.pdf]

Supplementary Table S3. Fungi – the 41 virtually restricted OTUs matched with T-RFs

Restr pattern	Phylum	Class	Order	Family
1	Ascomycota	Sordariomycetes	Sordariales	Lasiosphaeriaceae
2	Ascomycota	Dothideomycetes	Pleosporales	Venturiaceae
2	Ascomycota	Dothideomycetes	Pleosporales	Venturiaceae
2	Ascomycota	Dothideomycetes	Pleosporales	Venturiaceae
2	Ascomycota			
2	Ascomycota			
3	Ascomycota	Dothideomycetes	Pleosporales	Venturiaceae
3	Ascomycota	Eurotiomycetes	Onygenales	Onygenaceae
3	Ascomycota	Leotiomycetes	Helotiales	
3	Ascomycota	Leotiomycetes		
3	Ascomycota	Pezizomycotina_Incertae_sedis	Pezizomycotina_Incertae_sedis	Pezizomycotina_Incertae_sedis
3	Ascomycota	Pezizomycotina_Incertae_sedis	Pezizomycotina_Incertae_sedis	Pezizomycotina_Incertae_sedis
3	Ascomycota	Pezizomycotina_Incertae_sedis	Pezizomycotina_Incertae_sedis	Pezizomycotina_Incertae_sedis
3	Ascomycota	Pezizomycotina_Incertae_sedis	Pezizomycotina_Incertae_sedis	Pezizomycotina_Incertae_sedis
3	Ascomycota			
3	Ascomycota			
3	Ascomycota			
4	Basidiomycota	Tremellomycetes	Tremellales	Tremellales_Incertae_sedis
5	Ascomycota			
5	Ascomycota			
6	Ascomycota	Dothideomycetes	Pleosporales	Venturiaceae
7	Ascomycota	Dothideomycetes	Pleosporales	Lophiostomataceae
8	Ascomycota	Sordariomycetes	Hypocreales	Hypocreales_Incertae_sedis
9	Ascomycota	Sordariomycetes	Hypocreales	Nectriaceae
9	Ascomycota	Sordariomycetes	Hypocreales	
9	Ascomycota	Sordariomycetes	Hypocreales	
9	Ascomycota	Sordariomycetes		
10	Ascomycota	Sordariomycetes	Hypocreales	
10	Ascomycota	Sordariomycetes	Hypocreales	
11	Ascomycota			
11	Ascomycota			
12	Ascomycota	Leotiomycetes	Helotiales	Helotiales_Incertae_sedis
12	Ascomycota	Leotiomycetes	Helotiales	Helotiales_Incertae_sedis
12	Ascomycota	Leotiomycetes		
12	Ascomycota	Pezizomycotina_Incertae_sedis	Pezizomycotina_Incertae_sedis	Pezizomycotina_Incertae_sedis
12	Ascomycota	Pezizomycotina_Incertae_sedis	Pezizomycotina_Incertae_sedis	Pezizomycotina_Incertae_sedis
12	Ascomycota	Sordariomycetes		
12	Ascomycota			
12	Ascomycota			
13	Ascomycota			
14	Basidiomycota	Agaricomycetes	Atheliales	Atheliaceae
