# Supplementary material for: Trends in bacterial and fungal communities in ant nests observed with Terminal-Restriction Fragment Length Polymorphism (T-RFLP) and Next Generation Sequencing (NGS) techniques—validity and compatibility in ecological studies
Source: PeerJ. 2018 Jul 20;6:e5289. doi: 10.7717/peerj.5289 (PMC6055595; doi:10.7717/peerj.5289)
Supplement: Supplemental Information 1 [file peerj-06-5289-s008.pdf]

Bioinformatic analysis with UPARSE.

The analysis followed standard protocol for usearch/uparse (https://www.drive5.com/usearch/manual/).

1) Forward and reverse reads were first merged using the command fastq_merge pairs, allowing for a maximum of ten mismatches between reads, and a minimum percent ID of 10. Only merged reads with a length above 100 bp were retained for further analysis (primer and adaptor sequences were removed by the DNA Sequencing and Genomics Laboratory, Institute of Biotechnology, University of Helsinki).

2) Reads were filtered using the command -fastq_filter discarding reads with expected error scores below 1. Reads were not trimmed since this is not necessary with overlapping paired reads, since the reverse reads start at a primer-binding locus, and the merged sequence consequently ends at that locus. It should be noted that most reads discarded by quality filtering are only temporarily discarded and not lost from the analysis. All reads, including low-quality reads and singleton sequences, are used as input for making the OTU table. Quality-filtered reads are used as input for OTU clustering because otherwise, low-quality reads cause large numbers of spurious OTUs. Most low-quality reads successfully map to OTU sequences and are therefore recovered when the OTU table is made. This can be seen in the table S1, where the number of filtered reads is smaller than the number of reads in the final OTU table.

3) After filtering, reads were dereplicated with the command -derep_fulllength. Dereplicated reads were used as input for uparse, using the command cluster_otus. The cluster_otus command performs 97% OTU clustering, and removes chimeric sequences.

4) 97% OTUs were aligned to silva (bacterial reads) and unite (fungi) databases to determine taxonomic identity using the command -usearch_global. For taxonomic classification of the bacterial OTUs, the reference database RDP16s training set v.14 (Wang et al., 2007) was used, and for the fungal OTUs the RDP ITS Warcup training set v.4 (Deshpande et al., 2016) was used. The queries against the databases were done by using the RDP Naïve Bayesian Classifier with bootstrap cut-off at 80% (Wang et al., 2007).

5) The command usearch_global was then used to construct the final OTU table, with 97% similarity between OTUs, also using the reads discarded during the filtering step.

Several different pipelines exist for analysis of microbial communities (e.g. Schloss et al., 2009, Caporaso et al., 2010). The quality of the sequencing data, together with the choice of analysis pipeline and options chosen within these pipelines affect the final result (Knight et al., 2018). Overall, USEARCH/UPARSE performs well in comparison studies between analysis methods (Edgar, 2013), and appears to perform well the data in this study.

References

Caporaso JG., Kuczynski J., Stombaugh J., Bittinger K., Bushman FD., Costello EK., Fierer N., Peña AG., Goodrich JK., Gordon JI., Huttley GA., Kelley ST., Knights D., Koenig JE., Ley

RE., Lozupone CA., McDonald D., Muegge BD., Pirrung M., Reeder J., Sevinsky JR., Turnbaugh PJ., Walters WA., Widmann J., Yatsunenko T., Zaneveld J., Knight R. 2010. QIIME allows analysis of high-throughput community sequencing data. *Nature Methods* 7:335–336. DOI: 10.1038/nmeth.f.303.

Deshpande V., Wang Q., Greenfield P., Charleston M., Porras-Alfaro A., Kuske CR., Cole JR., Midgley DJ., Tran-Dinh N. 2016. Fungal identification using a Bayesian classifier and the Warcup training set of internal transcribed spacer sequences. *Mycologia* 108:1–5. DOI: 10.3852/14-293.

Edgar RC. 2013. UPARSE: highly accurate OTU sequences from microbial amplicon reads. *Nature methods* 10:996–8. DOI: 10.1038/nmeth.2604.

Knight R., Vrbanac A., Taylor BC., Aksenov A., Callewaert C., Debelius J., Gonzalez A., Kosciolek T., McCall LI., McDonald D., Melnik A V., Morton JT., Navas J., Quinn RA., Sanders JG., Swafford AD., Thompson LR., Tripathi A., Xu ZZ., Zaneveld JR., Zhu Q., Caporaso JG., Dorrestein PC. 2018. Best practices for analysing microbiomes. *Nature Reviews Microbiology* 16:410–422. DOI: 10.1038/s41579-018-0029-9.

Schloss PD., Westcott SL., Ryabin T., Hall JR., Hartmann M., Hollister EB., Lesniewski RA., Oakley BB., Parks DH., Robinson CJ., Sahl JW., Stres B., Thallinger GG., Van Horn DJ., Weber CF. 2009. Introducing mothur: open-source, platform-independent, community-supported software for describing and comparing microbial communities. *Applied and environmental microbiology* 75:7537–41. DOI: 10.1128/AEM.01541-09.

Wang Q., Garrity GM., Tiedje JM., Cole JR. 2007. Naive Bayesian classifier for rapid assignment of rRNA sequences into the new bacterial taxonomy. *Applied and environmental microbiology* 73:5261–7. DOI: 10.1128/AEM.00062-07.
